# Factors associated with sedentary behavior among community-dwelling breast cancer survivors aged 50 years or older

**DOI:** 10.1038/s41598-024-51172-x

**Published:** 2024-03-21

**Authors:** Jae Hyeon Park, Jung Soo Lee, Hyung Seok Nam, Yeo Hyung Kim

**Affiliations:** 1 Department of Rehabilitation Medicine, Hanyang University College of Medicine, Seoul, 04763 Republic of Korea; 2 Department of Rehabilitation Medicine, College of Medicine, The Catholic University of Korea, Seoul, 06591 Republic of Korea; 3 Department of Rehabilitation Medicine, Sheikh Khalifa Specialty Hospital, Ras al Khaimah, United Arab Emirates

**Keywords:** Health care, Medical research, Oncology, Risk factors

## Abstract

Although increased sedentary behavior is associated with poor health outcomes among breast cancer survivors, the factors associated with high sedentary time in community-dwelling breast cancer survivors are unknown. This study aimed to identify factors associated with sedentary behavior in Korean community-dwelling breast cancer survivors aged ≥ 50 years. We included 205 breast cancer survivors from the Korea National Health and Nutrition Examination Survey. Total daily sedentary time was evaluated using questions from the Korean version of the Global Physical Activity Questionnaire. We used complex-sample multivariable-adjusted logistic regression analyses to analyze the associations between sociodemographic factors, medical factors, and health-related quality of life and high sedentary time (≥ 420 min/day). Among the Korean community-dwelling breast cancer survivors, 48.2% had a high daily sedentary time. Insufficient aerobic exercise (OR 2.29; 95% CI 1.12–4.69), diabetes (OR 3.37; 95% CI 1.22–9.33), and unemployed status (OR 2.29; 95% CI 1.05–5.02) were independently associated with high sedentary time after the adjustment for multiple sociodemographic and medical confounders. Participants with a low sedentary time (< 420 min/day) showed a significantly higher mean European Quality of Life 5-Dimensions (EQ-5D) index than those with a high sedentary time after adjusting for multiple confounders (0.89 ± 0.03 vs. 0.82 ± 0.04; *P* = 0.001). Among the EQ-5D dimensions, problems in mobility (OR 3.37; 95% CI 1.42–7.98) and pain/discomfort (OR 2.64; 95% CI 1.24–5.63) dimensions showed positive associations with high sedentary time. Middle- or older-aged breast cancer survivors with insufficient aerobic exercise, diabetes, unemployed status, and impaired quality of life are more likely to have a high sedentary time. Reducing sedentary behavior in this population requires a tailored approach that considers diverse sociodemographic, medical, and quality-of-life factors.

## Introduction

Sedentary behavior refers to any waking behavior characterized by an energy expenditure ≤ 1.5 metabolic equivalents (METs) while in a sitting, reclining, or lying down posture^1^. Recent research suggests that passive standing, which cannot be classified as sedentary behavior under the current definition, should be categorized as upright behavior with an intensity ≤ 1.5 METs^2^. Sedentary behavior has emerged as a risk factor for detrimental health outcomes independent of physical activity^3,4^. Excessive sedentary time is associated with increased mortality, cardiometabolic risk, and mental health issues in adults^3^. Furthermore, among cancer survivors, sedentary behavior is correlated with impaired cardiovascular fitness and overall morality^5^. Therefore, the World Health Organization, American Society of Clinical Oncology, and American College of Sports Medicine recommend that cancer survivors minimize their sedentary time and replace it with physical activity^6–8^.

Sedentary behavior in cancer survivors has been actively studied for its clinical significance, as cancer survivors are reportedly more sedentary than individuals without cancer^9,10^. Furthermore, sedentary behavior reportedly does not vary among cancer types, whereas physical activity engagement does^9^. Sedentary behavior has been most commonly assessed in breast cancer survivors. Because breast cancer is the most common cancer in women and has an improved prognosis, the number of breast cancer survivors has increased^11,12^. Breast cancer survivors are more sedentary than healthy individuals without cancer^13,14^. This increased sedentary time in breast cancer survivors could be attributed to the cancer treatment side effects, fear of lymphedema, or concerns about exercise safety^15^. In addition, increased sedentary behavior is associated with poor health outcomes in breast cancer survivors, including increased fatigue, obesity, cardiovascular diseases, and higher mortality rates^16,17^.

Sedentary behavior is influenced by a combination of sociodemographic and medical factors, identifying these factors is essential for reducing sedentary behavior^4^. Despite the potential to improve health outcomes by identifying factors associated with sedentary behavior, few studies have investigated these factors in breast cancer survivors. Previous studies have reported that obesity, pain, increased comorbidities, more advanced disease state, employed status, and less physical activity are associated with excessive sedentary time, whereas marital status and higher income are not^18–21^. However, the reported correlates are inconsistent across studies. Furthermore, despite the increasing incidence of breast cancer in Asian countries and the influence of racial and sociocultural differences on physical activity behavior among cancer survivors, most earlier studies were primarily conducted on Caucasian and African American populations of breast cancer survivors aged ≥ 18 years in the United States or other Western countries^22–24^.

Although sedentary behavior is important from a clinical and public health perspective, the factors associated with a high sedentary time are not currently established in breast cancer survivors, particularly in community-dwelling Asians, including Koreans. Furthermore, considering the growing emphasis on reducing sedentary time in older adults^4,25^, it is essential to identify the factors associated with a high sedentary time in middle-aged and older breast cancer survivors. Therefore, this study aimed to identify the factors independently associated with a high sedentary time in community-dwelling Korean breast cancer survivors aged ≥ 50 years after adjusting for multiple sociodemographic and medical confounders.

## Methods

### Study design and participants

This study included participants recruited from KNHANES VI, VII, and VIII. The Korea Centers for Disease Control and Prevention (KCDC) annually collects information on health behaviors and the status of chronic diseases among community-dwelling residents through the KNHANES. The KNHANES introduced a multilevel, stratified, clustered probability sampling method to collect data representing the country’s population. The detailed survey design, variable definitions, and de-identified raw data are available on the website (https://knhanes.kdca.go.kr/). The protocol for KNHANES was approved by the Institutional Review Board (IRB) at KCDC, and informed consent was obtained from all participants. Since publicly available data was used, the IRB of our hospital waived the need for ethical approval. All analyses were conducted in compliance with the relevant guidelines.

Among the 47,309 community-dwelling people recruited between 2014 and 2019, 20,546 participants (8888 men and 11,658 women) aged ≥ 50 years were screened. Breast cancer survivors were defined as those who answered “yes” to the question “Have you been diagnosed with breast cancer by a doctor?”^22^. After excluding those with missing values, 205 female breast cancer survivors aged ≥ 50 years were included in the analysis.

### Variables

The Korean version of the Global Physical Activity Questionnaire (GPAQ) evaluated sedentary behavior and aerobic exercise^26^. The time spent sitting, excluding sleeping time, was assessed with the question: “How much time do you usually spend sitting or reclining on a typical day?”. Sitting at a desk, sitting with friends, traveling in a car, bus, or train, reading, playing cards, and watching television are examples of sedentary behavior. Individuals were classified as having “high sedentary time” at ≥ 420 min of daily sedentary time and as having “low sedentary time” at < 420 min of daily sedentary time^27^. The GPAQ also gathers the time spent in a typical week performing moderate and vigorous aerobic physical activities. A sufficient level of aerobic exercise was defined as spending ≥ 150 min/week of moderate-intensity aerobic activity, or ≥ 75 min/week of vigorous-intensity aerobic activity, or an equivalent combination of moderate- and vigorous-intensity physical activity according to the WHO recommendation^28^. Sufficient resistance exercise level was defined as performing resistance exercises, such as push-ups, sit-ups, dumbbells, weights, and barbells, ≥ 2 days in the past week^28^.

Breast cancer survivors were also asked whether they were currently receiving treatment for breast cancer. The years since breast cancer diagnosis were classified as < 5 years or ≥ 5 years considering the definition of long-term cancer survivors by the American Cancer Society^29,30^. Individuals with a systolic blood pressure ≥ 140 mmHg, a diastolic blood pressure ≥ 90 mmHg, or receiving antihypertensive drugs were considered those with hypertension. Individuals with HbA1c ≥ 6.5%, fasting blood glucose ≥ 126 mg/dL, diagnosis of diabetes by a doctor, use of hypoglycemic agents, or insulin injections were considered to have diabetes. Participants with hypercholesterolemia were regarded as those with total cholesterol ≥ 240 mg/dL or taking cholesterol-lowering drugs. A person with a hemoglobin level < 12 g/dL was considered to have anemia. Individuals diagnosed with depression by a doctor were believed to have depression. Participants were categorized as obese when their body mass index (BMI) was ≥ 25 kg/m^2^ according to the World Health Organization’s guidelines for obesity classification in the Asia–Pacific region^31^.

Health-related quality of life was evaluated using the three-level version of the EQ-5D (EQ-5D-3L)^30,32^. The EQ-5D-3L encompasses five dimensions (mobility, self-care, usual activities, pain/discomfort, and anxiety/depression) with three levels (no problems, some problems, and extreme problems) for each dimension. This study classified health-related problems as having some or extreme problems for each of the five dimensions^30,33^. The EQ-5D index was calculated, with a score of 1 indicating full health and 0 indicating a state as bad as death. Participants with a body mass index ≥ 25 kg/m^2^ were considered obese. Information on the participants’ residence (urban or rural), education level (> 9 years, ≤ 9 years), and occupational status (employed or unemployed) was also recorded.

### Statistical analysis

The characteristics of breast cancer survivors according to their sedentary behavior status were examined using a complex-sample chi-square test. Complex-sample Chi-square tests were also used to compare the prevalence of problems in each EQ-5D dimension by sedentary behavior status. Factors associated with a high sedentary time were evaluated using complex sample multivariable-adjusted logistic regression analyses. Complex-sample general linear models adjusted for potential confounders were used to compare the mean EQ-5D index according to sedentary behavior status. The independent associations between a high sedentary time and problems in each EQ-5D dimension were analyzed using complex-sample multivariable-adjusted logistic regression analyses. Since the KNHANES data are from a sample survey rather than a complete survey, the analyses are recommended to reflect the complex sample design to interpret the results as those of the entire Korean population. Therefore, we used the complex-sample procedures of SPSS version 24 (IBM SPSS Inc., Armonk, NY, USA) to apply sample weights that could adjust for the sampling errors, unequal probability sampling, and non-response errors of the KNHANES.

## Results

The weighted mean age of breast cancer survivors who participated in this study was 62.4 years (standard error [SE] 0.8). The prevalence of obesity, a rural residence, education ≤ 9 years, unemployed status, insufficient aerobic exercise, and insufficient resistance exercise among breast cancer survivors was 38.4%, 15.3%, 43.2%, 63.7%, 54.0%, and 84.1%, respectively. Among the participating breast cancer survivors, 58.9% had been diagnosed with breast cancer 5 or more years prior, while 29.9% were currently undergoing breast cancer treatment. The proportions of participants with hypertension, diabetes, hypercholesterolemia, anemia, and depression were 39.9%, 17.9%, 33.3%, 15.6%, and 8.4%, respectively.

The weighted mean total sedentary time of participants was 448.6 min/day (SE, 15.0), and the weighted prevalence of a high sedentary time was 48.2% (SE, 3.7%). As shown in Table 1, participants with a high sedentary time tended to be unemployed (71.7% vs. 56.2%, *P* = 0.034), engage in insufficient aerobic exercise (64.2% vs. 44.5%, *P* = 0.005), and have diabetes (24.5% vs. 11.8%, *P* = 0.028) than participants with a low sedentary time. However, participants with a high sedentary time had similar age, obesity status, residence, educational level, resistance exercise level, years since cancer diagnosis, current cancer treatments, hypercholesterolemia, anemia, or depression compared to those with a low sedentary time.

**Table 1:** Characteristics of community-dwelling breast cancer survivors by sedentary behavior status.

Variables	Participants with low daily sedentary time	Participants with high daily sedentary time	*P*
Unweighted number (n)	104	101	
Weighted number (n)	7633	8423	
Age (years)			0.844
50–59	48.2 (5.6)	45.3 (5.9)	
60–69	27.4 (4.6)	26.5 (4.2)	
≥ 70	24.4 (5.2)	28.1 (4.7)	
Obesity			0.118
No	67.8 (5.4)	55.0 (5.3)	
Yes	32.2 (5.4)	45.0 (5.3)	
Residence			0.596
Urban	83.6 (3.5)	85.9 (3.9)	
Rural	16.4 (3.5)	14.1 (3.9)	
Education			0.746
> 9 years	57.9 (4.8)	55.5 (5.3)	
≤ 9 years	42.1 (4.8)	44.5 (5.3)	
Occupation			0.034
Employed	43.8 (5.0)	28.3 (4.8)	
Unemployed	56.2 (5.0)	71.7 (4.8)	
Aerobic exercise			0.005
Sufficient	55.5 (4.7)	35.8 (5.2)	
Insufficient	44.5 (4.7)	64.2 (5.2)	
Resistance exercise			0.822
Sufficient	15.3 (4.1)	16.5 (3.6)	
Insufficient	84.7 (4.1)	83.5 (3.6)	
Years since cancer diagnosis			0.067
< 5 years	33.8 (5.3)	49.0 (5.6)	
≥ 5 years	66.2 (5.3)	51.0 (5.6)	
Currently on cancer treatment			0.200
No	74.6 (5.3)	65.3 (5.1)	
Yes	25.4 (5.3)	34.7 (5.1)	
Comorbidities			
Hypertension	37.9 (5.5)	42.1 (4.8)	0.570
Diabetes	11.8 (3.1)	24.5 (4.7)	0.028
Hypercholesterolemia	31.5 (5.5)	35.3 (5.8)	0.629
Anemia	15.8 (5.0)	15.4 (4.5)	0.947
Depression	8.4 (3.0)	8.4 (2.8)	0.993

Values are % (SE).

### Association of demographic and health-related factors with sedentary behavior

Table 2 shows the associations between demographic and health-related factors and sedentary time among Korean community-dwelling breast cancer survivors. Unemployed status (Odds ratio [OR] 1.97; 95% confidence intervals [CI] 1.00–3.88), insufficient aerobic exercise (OR 2.23; 95% CI 1.20–4.15), and diabetes status (OR 2.42; 95 CI 1.07–5.50) were significantly associated with a high sedentary time. These associations remained significant after multivariable adjustment. The breast cancer survivors who were unemployed (adjusted OR 2.29; 95% CI 1.05–5.02), engaged in insufficient aerobic exercise (adjusted OR 2.29; 95% CI 1.12–4.69), and had diabetes (adjusted OR 3.37; 95% CI 1.22–9.33) were more likely to have a high sedentary time even after adjusting for multiple potential confounders. Breast cancer-related variables, years since cancer diagnosis (adjusted OR 0.45; 95% CI 0.17–1.24), and currently undergoing cancer treatment (adjusted OR 0.78; 95% CI 0.30–2.00) were not associated with a high sedentary time after the adjustment for multiple confounders.

**Table 2:** Factors associated with a high daily sedentary time in breast cancer survivors.

Variables	Unadjusted OR (95% CI)	Adjusted OR* (95% CI)
Age (years)		
50–59	Reference	Reference
60–69	1.03 (0.48–2.17)	1.07 (0.42–2.70)
≥ 70	1.23 (0.54–2.76)	0.53 (0.16–1.72)
Obesity		
No	Reference	Reference
Yes	1.73 (0.84–3.53)	1.84 (0.76–4.45)
Residence		
Urban	Reference	Reference
Rural	0.84 (0.41–1.72)	0.71 (0.35–1.44)
Education		
> 9 years	Reference	Reference
≤ 9 years	1.10 (0.57–2.13)	1.01 (0.46–2.21)
Occupation		
Employed	Reference	Reference
Unemployed	1.97 (1.00–3.88)	2.29 (1.05–5.02)
Aerobic exercise		
Sufficient	Reference	Reference
Insufficient	2.23 (1.20–4.15)	2.29 (1.12–4.69)
Resistance exercise		
Sufficient	Reference	Reference
Insufficient	0.92 (0.40–2.09)	0.59 (0.25–1.39)
Years since cancer diagnosis		
< 5 years	Reference	Reference
≥ 5 years	0.53 (0.27–1.06)	0.45 (0.17–1.24)
Currently on cancer treatment		
No	Reference	Reference
Yes	1.56 (0.78–3.12)	0.78 (0.30–2.00)
Comorbidities		
Hypertension	1.19 (0.63–2.24)	0.88 (0.38–2.04)
Diabetes	2.42 (1.07–5.50)	3.37 (1.22–9.33)
Hypercholesterolemia	1.19 (0.57–2.46)	0.98 (0.43–2.28)
Anemia	0.97 (0.34–2.77)	0.99 (0.24–4.04)
Depression	1.00 (0.99–2.92)	0.83 (0.28–2.47)

Values are OR (95% CI).

*Adjusted for all other variables in the columns.

OR, Odds ratio; CI, Confidence intervals.

### Association of quality of life with sedentary behavior

**Figure 1:**
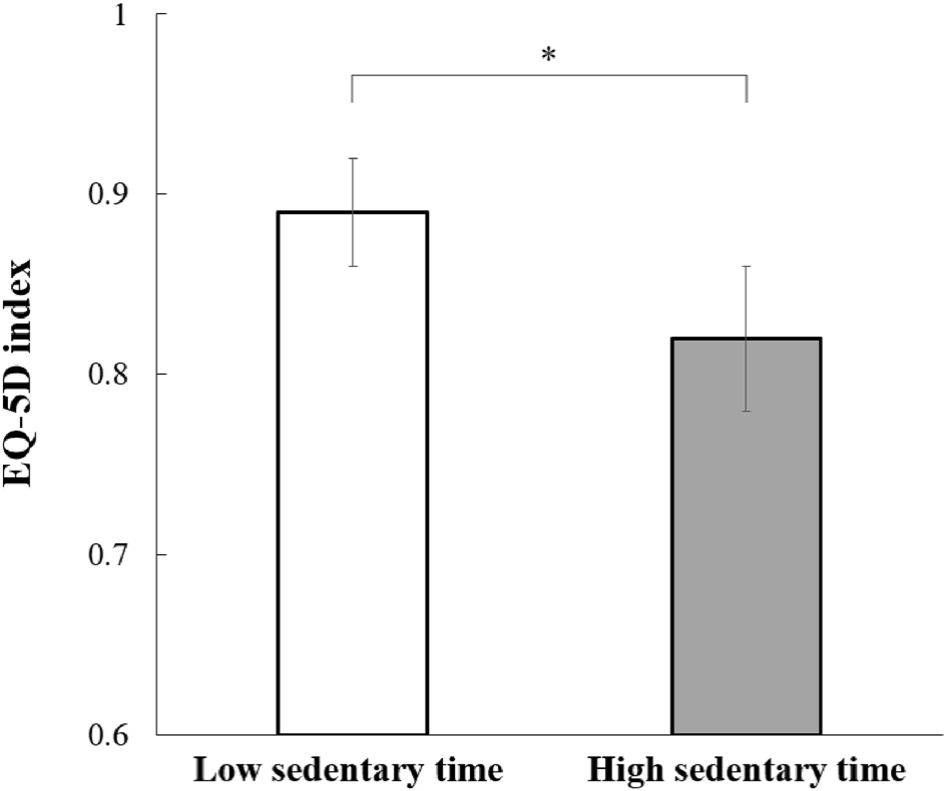
Adjusted mean EQ-5D index by sedentary behavior status. Values are adjusted for age, obesity, residence, education, occupation, aerobic exercise, resistance exercise, years since cancer diagnosis, currently on cancer treatment, hypertension, diabetes, hypercholesterolemia, anemia, and depression. *P < 0.05.

**Figure 2:**
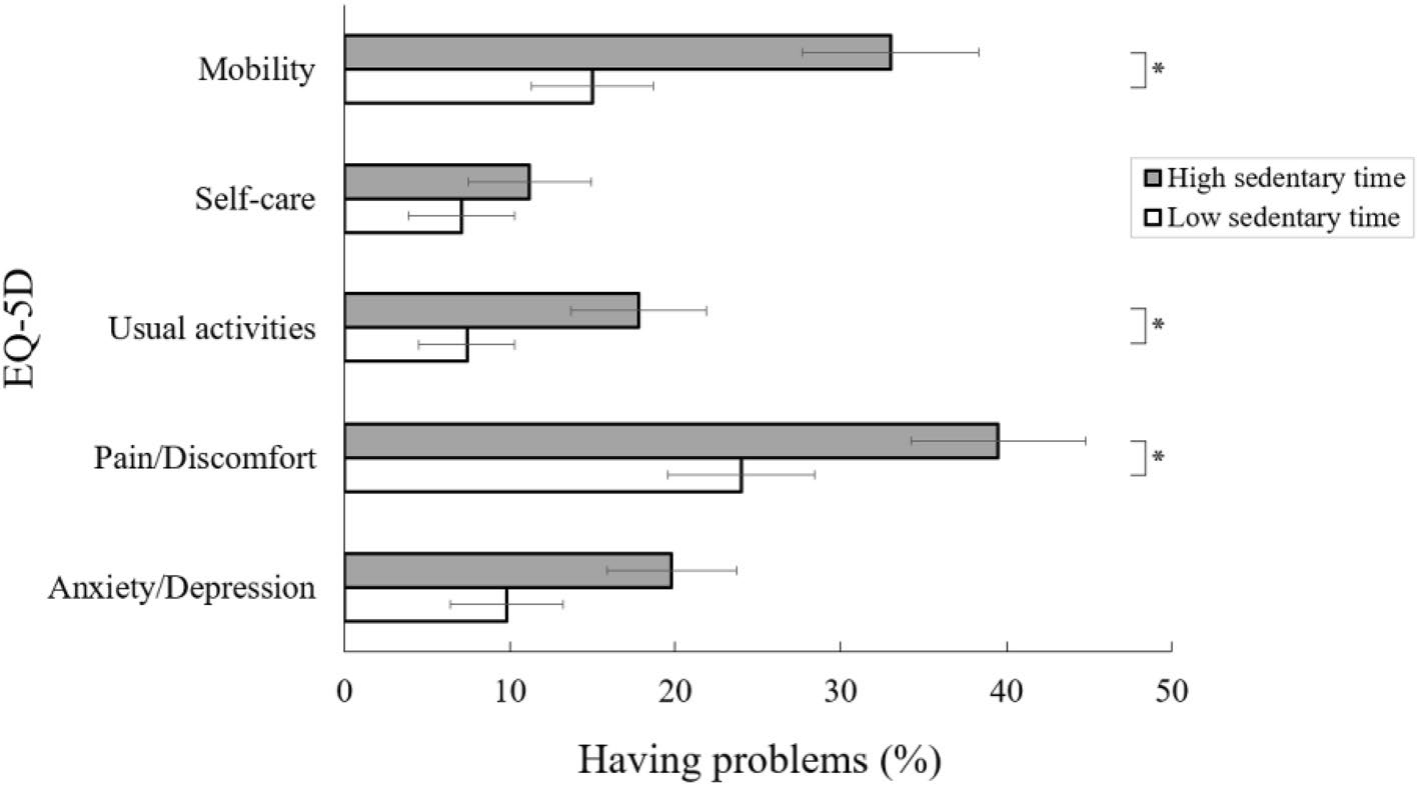
Weighted prevalence of having problems in each EQ-5D dimension according to sedentary behavior status among breast cancer survivors. *P < 0.05.

Figure 1 shows that the adjusted mean EQ-5D index was significantly higher in participants with a low sedentary time than in those with a high sedentary time (0.89 ± 0.03 vs. 0.82 ± 0.04; *P* = 0.001). Figure 2 shows the weighted prevalence of problems in each EQ-5D dimension according to sedentary behavior status among Korean breast cancer survivors. Breast cancer survivors with a high sedentary time were significantly more likely to have problems with mobility (33.0% vs. 15.0%, *P* = 0.007), usual activities (17.8% vs. 7.4%, *P* = 0.046), and pain/discomfort (39.5% vs. 24.0%, *P* = 0.019) than those with a low sedentary time. The prevalence of problems in the self-care and anxiety/depression dimensions did not differ according to sedentary time among breast cancer survivors. The associations between a high sedentary time and problems in each EQ-5D dimension are shown in Table 3. After adjusting for multiple confounders, the breast cancer survivors who had problems in mobility (adjusted OR 3.37; 95% CI 1.42–7.98) and pain/discomfort (adjusted OR 2.64; 95% CI 1.24–5.63) dimensions tended to have a high sedentary time than those who had no problems in these dimensions.

**Table 3:** Adjusted ORs for a high daily sedentary time in breast cancer survivors.

EQ-5D dimension	No problems	Having problems*	*P*
Mobility	Reference	3.37 (1.42–7.98)	0.006
Self-care	Reference	2.27 (0.64–8.10)	0.203
Usual activity	Reference	3.14 (0.89–11.01)	0.074
Pain/discomfort	Reference	2.64 (1.24–5.63)	0.013
Anxiety/depression	Reference	2.81 (0.74–10.71)	0.128

Values are OR (95% CI).

*Adjusted for age, obesity, residence, education, occupation, aerobic exercise, resistance exercise, years since cancer diagnosis, current cancer treatment, hypertension, diabetes, hypercholesterolemia, anemia, and depression.

## Discussion

This nationally representative study demonstrated that insufficient aerobic exercise, diabetes, and unemployment were independently associated with a high sedentary time in Korean middle-aged and older community-dwelling breast cancer survivors. Furthermore, a worsened health-related quality of life was associated with a high sedentary time in breast cancer survivors. Importantly, breast cancer survivors with mobility and pain/discomfort problems are more likely to have a high sedentary time. Korean community-dwelling breast cancer survivors aged ≥ 50 years with insufficient aerobic exercise, unemployment, diabetes, and issues of mobility and pain/discomfort tended to be more sedentary. Therefore, clinicians should focus more on sedentary behavior in Korean community-dwelling breast cancer survivors by considering factors such as pain, poor mobility, diabetes, and unemployed status.

Herein, breast cancer survivors who engaged in insufficient aerobic exercise showed 2.29-fold higher odds of having a high sedentary time than those who performed sufficient aerobic exercise. Our findings are consistent with previous studies that found sedentary breast cancer survivors less physically active^20^. A previous study in the United States involving breast cancer survivors aged 21–85 years who received an aerobic exercise intervention found that it led to a reduction in their total sedentary time and an increase in moderate-to-vigorous physical activity, which supports our findings^34^. However, another randomized controlled trial in the United States reported that exercise interventions that did not specifically target reduction in sedentary behavior did not alter sedentary behavior in breast cancer survivors aged ≥ 21 years^35^. Although the present study demonstrated an independent association between insufficient aerobic exercise and a high sedentary time in Korean breast cancer survivors, more research is needed on whether longitudinal improvement of both can be achieved by controlling one of them.

Among the health-related quality of life dimensions, problems of mobility and pain/discomfort showed an independent positive association with a high sedentary time. The self-care and anxiety/depression dimensions did not show a significant association with a high sedentary time. However, after adjusting for multiple confounders, the association between problems in the usual activity dimension and a high sedentary time was eliminated, implying that this association was influenced by sociodemographic and medical factors. Our findings are similar to those of Hartman et al.^36^, who reported that total sedentary time was associated with a worse physical but not mental quality of life in breast cancer survivors. Although the overall health-related quality of life is associated with sedentary behavior, it is necessary to recognize the context of quality of life considering the dimension-specific association between quality of life and sedentary behavior.

Previous studies suggesting that sedentary behavior was associated with pain in breast cancer survivors also support our findings^21,37,38^. Breast cancer survivors often experience pain for various reasons, and pain is common with treatments such as aromatase inhibitors^11^. As this was a cross-sectional study, a cause-and-effect relationship could not be established, and it appears that managing pain may contribute to reducing daily sedentary time. Thus, reducing pain among breast cancer survivors is important for improving quality of life, decreasing sedentary time, and continuing treatment, which can improve prognosis^11^.

Our study showed that diabetes was independently associated with a high sedentary time in Korean community-dwelling breast cancer survivors. A prospective online questionnaire study revealed that increased comorbidities were associated with prolonged sedentary behavior in breast cancer survivors^19^. However, the previous studies did not analyze the associations of each comorbidity with increased sedentary behavior. To the best of our knowledge, studies suggesting an independent association between diabetes and a high sedentary time among breast cancer survivors are limited. Since diabetes is an established predictor of mortality in breast cancer survivors, the independent association between diabetes and sedentary behavior in this population has important clinical implications^39^. Community-based programs to reduce sedentary behavior in breast cancer survivors diagnosed with diabetes are needed, and screening for diabetes in breast cancer survivors is essential.

In this study, unemployed breast cancer survivors were more likely to have higher daily sedentary time than employed breast cancer survivors. Our findings contradict those of a previous study in the USA that reported that working at least part-time was associated with prolonged non-leisure and weekday sitting^18^. The previous study included young breast cancer survivors aged ≥ 18 years, whereas our study included middle-aged or older breast cancer survivors. Therefore, the discordant age distribution of the participants between these two studies may have contributed to the reversed association between occupational status and sedentary time. Additionally, because work-related sedentary time differs depending on the type of occupation, the association between occupation and sedentary time may be influenced by the country and culture.

Herein, Korean community-dwelling breast cancer survivors aged ≥ 50 years wither more daily sedentary time exhibited a significantly poorer health-related quality of life than those with less daily sedentary time. Inconsistencies persist in the results of previous studies on the association between sedentary behavior and quality of life. Some studies reported that increased sedentary behavior is associated with poor quality of life^36,40,41^, while others have reported no association between sedentary behavior and quality of life^17,42,43^. Earlier studies that reported no association between sedentary behavior and quality of life have been conducted mostly in Western countries^17,42,43^, and were conducted with newly diagnosed breast cancer patients^43^ or included young breast cancer survivors aged ≥ 18 years^17,43^. Therefore, different age groups, countries, cultures, and breast cancer statuses across studies may have contributed to these inconsistent results.

Approximately half of the Korean community-dwelling breast cancer survivors spent ≥ 420 min of daily sedentary time. Furthermore, the mean total sedentary time of 448.6 min/day observed in this study was similar to those of previous studies assessing the total sedentary time in breast cancer survivors^13,14,44^. Earlier studies have estimated that breast cancer survivors are sedentary for approximately 66–78% of their waking time^14,45^. Given that sedentary behavior is a known risk factor for poor health outcomes in breast cancer survivors, half of the community-dwelling breast cancer survivors require public health intervention. Furthermore, because increased sedentary behavior is independently associated with insufficient aerobic exercise, unemployment, and diabetes, more attention should be paid to breast cancer survivors with these factors.

The merit of this study is that it documents the factors associated with a high sedentary time in Korean community-dwelling breast cancer survivors, which has not previously been reported. To adjust to physical activity, one must have a thorough understanding of the factors that contribute to sedentary behavior. There are limitations to this study. First, the causal relationship between the identified factors and sedentary behavior could not be determined owing to the cross-sectional design of the study. Although we used nationally representative data collected over 6 years, the number of community-dwelling breast cancer survivors remained modest. The results can be interpreted as representative of all non-hospitalized Korean breast cancer survivors; however, generalization to other countries and cultures is inappropriate. Furthermore, information on the stage, severity, treatment type, and time since treatment for breast cancer was not surveyed in the Korea National Health and Nutrition Examination Survey (KNHANES); therefore, the effects of these factors could not be analyzed. Recall bias could be present because sedentary behavior was investigated using a self-reported questionnaire.

In conclusion, our study demonstrated that a high sedentary time is associated with insufficient aerobic exercise, diabetes, unemployment, and poor quality of life in Korean community-dwelling breast cancer survivors. Future longitudinal studies are needed to elucidate the cause-effect relationships between these factors and sedentary behavior. Although the causal relationships are unclear, breast cancer survivors who engage in insufficient aerobic exercise, have diabetes, or are unemployed are more likely to have high daily sedentary time. Therefore, people with these factors deserve public health attention. Targeted programs are required to reduce the sedentary time in this population.

## Data Availability

The data analyzed in the present study are publicly available from the KNHANES website (https://knhanes.kdca.go.kr/).
